# Excision of the Gluteal Cleft Pilonidal Sinus, Its Track, and the Sudoriferous Gland Area En-bloc with Primary Repair in the Management of this Disease

**DOI:** 10.7759/cureus.2806

**Published:** 2018-06-14

**Authors:** Muhammad Hamza, Irfan Ahmed Nadeem, Tahira Yasmeen, Noor Fatima

**Affiliations:** 1 Surgery, Al Noor Surgery Hospital, Chakwal, PAK; 2 Gynecology and Obstetrics, Holy Family Hospital, Rawalpindi, PAK

**Keywords:** gluteal cleft, pilonidal sinuses, sudoriferous glands, primary repair

## Abstract

Introduction

Pilonidal disease is a common disease mostly affecting young males with a significant reduction in their working capabilities, hence, ideal and simple management of this disease is very important. Our study objective was to assess the recurrence rate of pilonidal sinus disease in patients with a complete excision of the pilonidal sinus, its track, and the sudoriferous gland area en-bloc with primary repair.

Methods

This descriptive study was conducted at Al-Noor Surgery, Chakwal, Pakistan, from February 2015 to July 2017. All patients who presented with acute and chronic pilonidal disease in the natal cleft, irrespective of age and gender, were included. We excluded from the study patients who had asymptomatic or recurrent pilonidal disease, previous pilonidal surgery, patients belonging to American Society of Anesthesiologists (ASA) Class III or above, immunodeficient patients, patients having bleeding disorders, patients on chemotherapy, and those diagnosed with comorbidities, e.g., anemia, tuberculosis, diabetes, and liver disease. Data were analyzed using Statistical Package for the Social Sciences (SPSS) version 21 (IBM Corp., Armonk, NY, US).

Results

During the study period, a total of one 112 patients were included. The male-to-female ratio was 55:1. The most common age group was the 21-25 age group. Chronic pilonidal disease was the most common presentation. Mean operative time was 22.09±3.65 minutes. There were no complications like bleeding, hematoma, edema, infection, and wound dehiscence. There was no recurrence in the six months follow-up. Hospital stay was 3.13±0.62 hours.

Conclusion

Simple excision of acute and chronic pilonidal sinus, its track, and the linked sudoriferous gland area en-bloc, followed by primary repair, is an effective approach to deal with this pathology.

## Introduction

Pilonidal sinus disease (PSD) is a common infection of the skin in the gluteal cleft, with a prevalence of 0.7% in the general population, more commonly affecting males (male to female ratio: 4:1) between the ages of 15 and 38 years [1-4]. Sex hormones act on sudoriferous glands and affect hair growth; other factors like sitting for long hours, excessive sweating, increased hair density, and obesity are collectively responsible for this disease [2,5]. Patients either may be asymptomatic or may present with acute pilonidal abscess, chronic fistula form, or a recurrent, complex pilonidal sinus disease [3]. The more common chronic disease is associated with severe long-term morbidity with the restraining of day-to-day activities, thereby leading to work loss and a significant socioeconomic impact on affected individuals. Thus, the significance of its effective management cannot be undermined [1-2,5-6].

The successful management of pilonidal sinus disease depends on principles like the simplicity of the procedure and limited hospital stays, with an early resumption of routine activities and no residual pain, complication, and recurrence [1,4]. At present, the surgical management options include the surgical excision of the pilonidal sinus with primary or secondary healing, Karydaki's flap reconstruction, and Limberg's flap transposition. However, controversies regarding ideal surgical management are common, with each method having its own pros and cons [7]. In our study, we completely excised pilonidal sinus, its track, and the linked sudoriferous gland area en-bloc and closed the wound. Since sudoriferous glands are responsible for this disease, we hypothesized that removal of these glands from the natal cleft would effectively eradicate this disease with no recurrence. Our study objective was to assess the recurrence rate of pilonidal sinus disease in patients with the effective removal of pilonidal sinus, along with the area containing the linked sudoriferous glands.

## Materials and methods

This study was carried out in Chakwal, Pakistan, from February 2015 to July 2017. It is a descriptive study and the sampling technique was nonprobability consecutive sampling. The sample size was calculated as 45 patients, keeping in consideration a 95% level of significance, an absolute precision of 5%, and a recurrence rate of 3% [7-8]. However, we included all the 112 patients who presented with pilonidal disease to this private setup during the study period.

We included patients who had acute and chronic pilonidal disease in the natal cleft of the gluteal area irrespective of age and gender. The diagnosis of pilonidal sinus disease was clinical. We excluded patients who had asymptomatic or recurrent pilonidal disease, previous pilonidal surgery, patients having ASA Class III or above, immunodeficient patients, patients having bleeding disorders, patient on chemotherapy, and those diagnosed to have comorbidities, e.g., anemia, tuberculosis, diabetes, and liver disease, from the study.

After consent and counseling regarding the study, preoperative routine investigation and pre-operative anesthetic assessment of all the patients were performed. All procedures were done under spinal anesthesia. Local anesthesia (a mixture of lidocaine, streptomycin, and low-dose long-acting steroids) was also given at the operative site at the start of the procedure to all the patients. Intravenous (IV) antibiotics (penicillin 500 mg and metronidazole 500 mg) were given to all patients after the test dose at the time of induction. Under aseptic measures, the operating area was shaved and painted with pyodine solution. The probe was inserted from the pilonidal sinus to the external opening of the sudoriferous glands in the natal cleft, as shown in Video 1, which delineated all the linked tracks.

**Video 1:** Probe examination of pilonidal sinus

The successful passing of the probe confirmed the presence of direct communication between the pilonidal sinus and sudoriferous gland. The overlying skin, all the tracks with their contents, the openings of the sinus and an area containing the sudoriferous glands, especially in the downward direction, were minimally excised. The hemostasis was secured with artery forceps or, if needed, with a vicryl 3/0 or 4/0 stitch. We didn’t use diathermy. We washed the wounds generously with normal saline, coapted the subcutaneous tissue and skin very accurately with vicryl 2/0, and left no dead space. Skin closure was done with proline 1/0. The drain was placed through the wound and kept in for two hours in all the patients. The dressing of the wound was done. All participants were called for follow-up after one and two weeks of surgery. The stitches were removed at two weeks after surgery in all the patients. The pain was assessed using the visual analog scale at the time of the patients' discharge. Operative time was the time from incision to the closure of the wound. Hospital stay was the time of admission of the patients to their discharge. Selective shaving/hair trimming three weeks post-operative was advised to all the patients. Postoperative antibiotics (tablet cefradine 500 mg TDS and tablet ofloxacin 200 mg BD) was advised for two weeks. Telephonic follow-up was done at six months after surgery.

Data were entered and analyzed using Statistical Package for the Social Sciences (SPSS) version 21 (IBM Corporation, Armonk, NY, US) [9]. All categorical variables are presented as frequencies and percentages whereas means along with standard deviations were calculated for numerical data.

## Results

Among the total of one 112 patients, 110 patients (98.21%) were males and two (1.79%) were females, with a male to female ratio of 55:1. The age group of the study participants is given in Table 1.

**Table 1:** Age groups of study participants having pilonidal disease

Age Groups ( Years )	Number	Percentage
16-20	23	20.5%
21-25	53	47%
26-30	10	9%
31-35	12	11%
36-40	6	5.5%
41-45	8	7%
Total	112	100%

The number and percentage of study participants having acute and chronic pilonidal sinus disease are shown in Table 2.

**Table 2:** Frequency and percentage of study participants having acute and chronic pilonidal sinus disease

Clinical Presentation of Disease	Number	Percentage
Acute presentation	22	19.64%
Chronic presentation	90	80.36%
Total	112	100%

During the surgical procedure, probing was successful in all the patients. As depicted in Table 3, multiple openings of the sudoriferous gland were seen in a majority of the subjects.

**Table 3:** Frequency and percentage of study participants having single or multiple external openings of sudoriferous glands

Number of External Openings of Sudoriferous Glands	Number of Patients	Percentage of Patients
1	2	1.78%
2	2	1.78%
3	106	94.66%
4	1	0.89%
5	1	0.89%
Total	112	100%

The mean operative time was 22.09±3.65 minutes. There were no complications like hematoma, infection, and wound dehiscence in any of the study participants. At the time of discharge, none of the study participants complained of pain. There was no recurrence at the six-month follow-up. The mean hospital stay was 3.13±0.62 hours.

## Discussion

**Figure 1:**
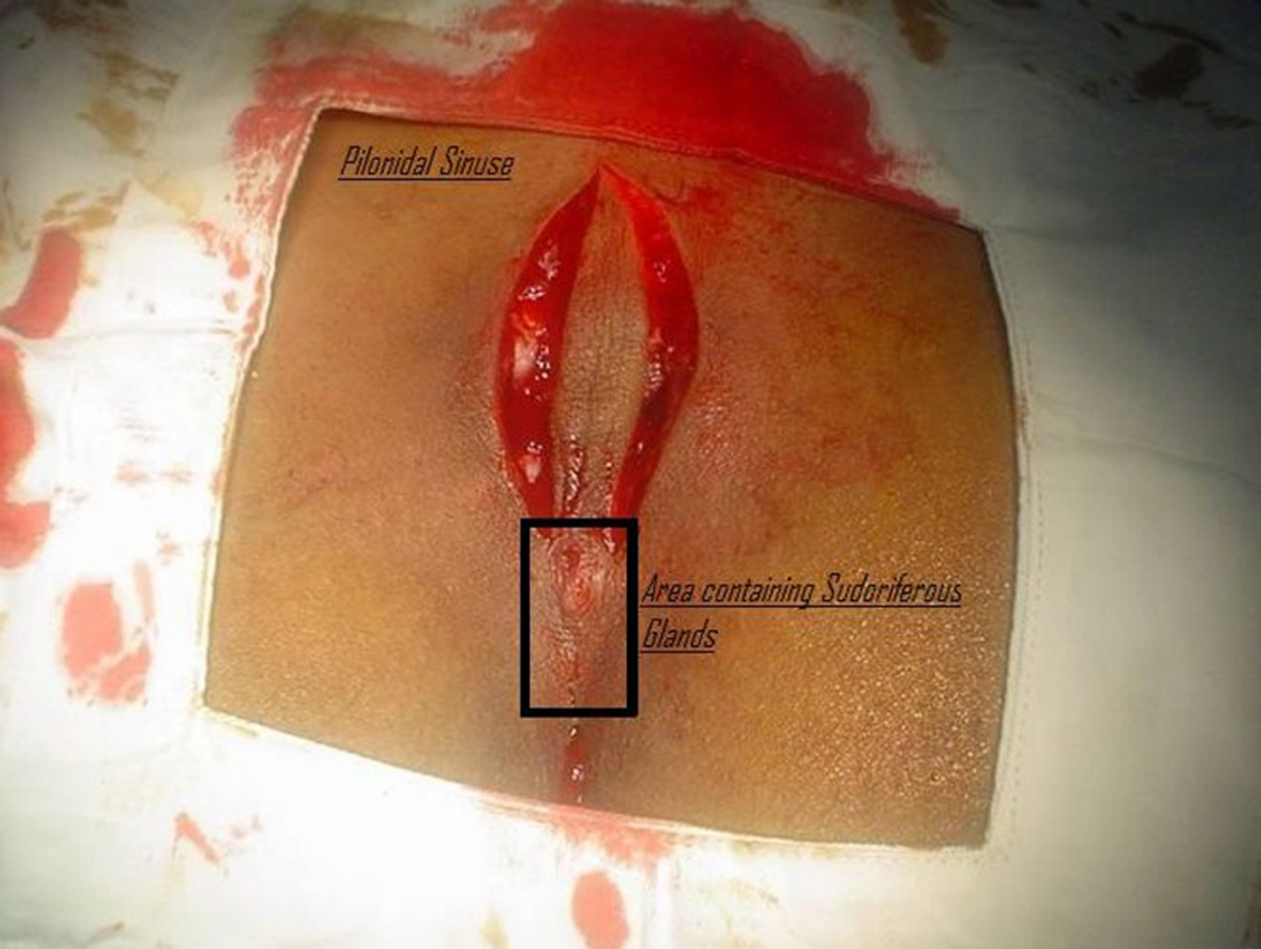
The black box shows the area of sudoriferous glands. This area should also be excised in pilonidal sinus surgery to avoid recurrence.

Pilonidal sinus disease (PSD) is an infection of the skin in the inter-gluteal cleft overlying the sacrum and coccyx, a short distance above the anus. It is characterized by one or more midline openings and sinus, which communicate with each other; these are lined by granulation tissue and contain dead hairs lying loosely within the lumen [1,3-5]. The role of sudoriferous glands and dead hair is well-described in the literature, as a major culprit responsible for this disease [1,4-5]. We are of the opinion that the removal of pilonidal sinuses, its track, and especially these sudoriferous glands area, as shown in Figure 1 should be our prime goal of pilonidal sinus disease surgery in order to avoid future recurrences of this disease.

In our study, we clearly depict that there is a direct communication between the pilonidal sinus and the opening of sudoriferous glands and that the effective removal of all these entities resulted in no recurrence. We are of the opinion that these openings of the sudoriferous glands serve as a gateway for hair in this area to enter into the subcutaneous tissue and collect there, producing an inflammatory response with a super-added infection leading to abscess formation, which bursts on the skin, leading to sinus formation.

The management of the pilonidal sinus disease is controversial and includes various conservative and surgical treatment options, but, many a time, it leads to unsatisfactory results [7,10-12]. The ideal treatment option should be of low cost, with a shorter admission time, no recurrence, minimal inconvenience, and less time off work [7,10-11]. Surgical techniques include incision and drainage, marsupialization, excision, and healing by primary or secondary intention and the techniques involve various flap procedures [7,12]. While there are studies suggesting that outcomes from flap repair are better, other studies suggest that the primary closure method could have a recur­rence rate of zero [12-13]. Flap closure has a low recurrence rate, but is a complex technique associated with greater blood loss and prolonged operative time; additionally, there is a potential risk of infection with a loss of flap [1,12,14]. The most commonly used surgical method for pilonidal sinus disease is excision with primary closure or secondary intention because it is simple, cost-effective, and has good results [12]. Nevertheless, excision with secondary intention is associated with the patients’ discomfort and prolonged hospital stay because the wound remains open for a long time while an excision with a primary closure is commonly associated with high recurrence and infection [10-12]. However, some studies suggest that infection rates are nearly equal in primary and secondary healing [1,10]. The recurrence rate after primary closure is the most important limiting factor for this method [11-12]. In literature, a recurrence rate of 0%-42% has been reported; but in our study, there was no recurrence [3,11-13]. In our opinion, the major factors leading to recurrence are the inadequate removal of pilonidal sinuses, its track, and especially the linked sudoriferous gland. Hence, complete excision with primary closure is important for the effective management and early discharge of patients, as shown in our study.

The hygienic postoperative shaving of hair around the natal cleft and its surrounding region is also emphasized by our study and in the literature [1,10,12]. In our study, none of the patients developed a postoperative hematoma. Other studies report 2.7%-4% postoperative bleeding, which was greater than in our study [7,11]. The explanation behind this was that there was no dead space left at the operation site [13]. The dead space at the operation site and subcutane­ously with/or without skin tension are the major causes of complications such as hematoma, infection, recurrence, and dehiscence [13].

In our study, we have delineated the track between pilonidal sinuses and the opening of the sudoriferous gland by using a probe because the value of using methylene blue to diffuse through the track is limited and questionable. Methylene dye does not completely diffuse through the tracts [1]. In our study, a majority of the patients had multiple linked external opening sudoriferous glands, which allow the probe to pass. This is very important, as identification and removal can limit the future recurrence of this disease.

The limitation of our study was that the data of a single-center study could not be globalized. Correct epidemiological data regarding the prevalence of this disease in the subcontinent are limited because of a social bias. The follow-up period for the recurrence of pilonidal sinus disease was short and long-term follow up is required.

## Conclusions

Pilonidal sinus is always linked with single or multiple sudoriferous glands. In order to completely remove this pathology and to avoid future recurrence, we need to address both of these entities simultaneously. A simple excision of pilonidal sinus, its track, and all the linked sudoriferous glands followed by primary repair is a good approach to deal with this pathology effectively.
